# Risk Factors for Late-Stage HIV Disease Presentation at Initial HIV Diagnosis in Durban, South Africa

**DOI:** 10.1371/journal.pone.0055305

**Published:** 2013-01-28

**Authors:** Paul K. Drain, Elena Losina, Gary Parker, Janet Giddy, Douglas Ross, Jeffrey N. Katz, Sharon M. Coleman, Laura M. Bogart, Kenneth A. Freedberg, Rochelle P. Walensky, Ingrid V. Bassett

**Affiliations:** 1 Division of Infectious Diseases, Massachusetts General Hospital, Boston, Massachusetts, United States of America; 2 Division of General Medicine, Massachusetts General Hospital, Boston, Massachusetts, United States of America; 3 Medical Practice Evaluation Center, Massachusetts General Hospital, Boston, Massachusetts, United States of America; 4 Department of Medicine, Brigham and Women's Hospital, Boston, Massachusetts, United States of America; 5 Department of Orthopedic Surgery, Brigham and Women's Hospital, Boston, Massachusetts, United States of America; 6 Division of Rheumatology, Brigham and Women's Hospital, Boston, Massachusetts, United States of America; 7 Center for AIDS Research, Harvard Medical School, Boston, Massachusetts, United States of America; 8 Department of Pediatrics, Harvard Medical School, Boston, Massachusetts, United States of America; 9 Departments of Epidemiology and Health Policy and Management, Harvard School of Public Health, Boston, Massachusetts, United States of America; 10 Department of Biostatistics, Boston University School of Public Health, Boston, Massachusetts, United States of America; 11 Department of Epidemiology, Boston University School of Public Health, Boston, Massachusetts, United States of America; 12 Data Coordinating Center, Boston University School of Public Health, Boston, Massachusetts, United States of America; 13 Boston Children's Hospital, Boston, Massachusetts, United States of America; 14 Department of Medicine, McCord Hospital, Durban, South Africa; 15 Department of Medicine, St. Mary's Hospital, Durban, South Africa; Alberta Provincial Laboratory for Public Health/ University of Alberta, Canada

## Abstract

**Background:**

After observing persistently low CD4 counts at initial HIV diagnosis in South Africa, we sought to determine risk factors for late-stage HIV disease presentation among adults.

**Methods:**

We surveyed adults prior to HIV testing at four outpatient clinics in Durban from August 2010 to November 2011. All HIV-infected adults were offered CD4 testing, and late-stage HIV disease was defined as a CD4 count <100 cells/mm^3^. We used multivariate regression models to determine the effects of sex, emotional health, social support, distance from clinic, employment, perceived barriers to receiving healthcare, and foregoing healthcare to use money for food, clothing, or housing (“competing needs to healthcare”) on presentation with late-stage HIV disease.

**Results:**

Among 3,669 adults screened, 830 were enrolled, newly-diagnosed with HIV and obtained a CD4 result. Among those, 279 (33.6%) presented with late-stage HIV disease. In multivariate analyses, participants who lived ≥5 kilometers from the test site [adjusted odds ratio (AOR) 2.8, 95% CI 1.7–4.7], reported competing needs to healthcare (AOR 1.7, 95% CI 1.2–2.4), were male (AOR 1.7, 95% CI 1.2–2.3), worked outside the home (AOR 1.5, 95% CI 1.1–2.1), perceived health service delivery barriers (AOR 1.5, 95% CI 1.1–2.1), and/or had poor emotional health (AOR 1.4, 95% CI 1.0–1.9) had higher odds of late-stage HIV disease presentation.

**Conclusions:**

Independent risk factors for late-stage HIV disease presentation were from diverse domains, including geographic, economic, demographic, social, and psychosocial. These findings can inform various interventions, such as mobile testing or financial assistance, to reduce the risk of presentation with late-stage HIV disease.

## Introduction

South Africa has more HIV-infected people than any other country, and over 1.4 million South Africans are now receiving antiretroviral therapy (ART) [1], [2]. However, despite this progress, 45–51% of ART-eligible people are still not receiving treatment [1], [3]. Not only do these HIV-infected people constitute nearly one-quarter of all AIDS-related deaths in sub-Saharan Africa, but they also transmit HIV to others [1]. Furthermore, late-stage HIV disease at initial HIV diagnosis has been associated with poor treatment response rates and higher mortality [4]–[11]. In August 2011, the South African Department of Health increased the ART initiation threshold from CD4<200 to <350 cells/mm^3^ to help reduce AIDS-related deaths [12]. However, the median CD4 count at the time of ART initiation was 111 cells/mm^3^ in a recent large South African cohort [4].

Increasing the CD4 count treatment threshold will have little benefit if the majority of people continue to present with dangerously low CD4 counts and late-stage HIV disease. Strategies to reduce AIDS-related mortality and decrease HIV transmission must include earlier diagnosis of HIV [13], which has been shown to improve survival [14]. Intensified efforts to promote early diagnosis of HIV-infected people in resource-limited settings are needed, but little is known about how to best target people in HIV testing campaigns [15], [16]. We conducted a large, prospective study to assess both the real and perceived barriers to presenting for HIV care in South Africa.

## Methods

### Sites and participants

We studied adults who presented for voluntary HIV counseling and testing at four outpatient clinical sites in Durban from August 2010 to November 2011. McCord Hospital is an urban, state-aided general hospital that serves the greater Durban area. St. Mary's Hospital in Mariannhill is a state-aided general hospital that serves a resource-limited population in a peri-urban area of Durban. Both McCord Hospital and St. Mary's Hospital have high-volume outpatient HIV clinics that have been providing ART since 2001 and 2003, respectively, and receiving President's Emergency Plan for AIDS Relief (PEPFAR) support since 2004. The other two sites, Tshelimnyama and Marianridge, are municipal primary health clinics located within the catchment area of St. Mary's Hospital. Throughout the course of the study, all four outpatient sites offered free HIV counseling and testing during normal working business hours.

We offered enrollment to all adults ≥18 years of age prior to HIV counseling and testing. We excluded those already known to be HIV-infected, pregnant, or unwilling to share their HIV test result with the research team. HIV testing, as well as participation in the study, carried no financial costs to the participant. All participants provided written informed consent either in English or Zulu. The study was approved by the ethics committees of McCord Hospital and St. Mary's Hospital in Durban, and Partners HealthCare (Protocol #: 2006-P-001379) in Boston.

### Data collection

We asked participants about personal demographics, proximity to the HIV clinic, and prior healthcare usage and HIV testing. We recorded responses to 12 questions related to perceived personal barriers for seeking HIV testing and medical care during the prior 6 months [17]. We asked 5 questions about emotional health over the previous month with each response rated on a 6-point Likert scale (ranging from 1 being “all of the time” or “always” to 6 being “none of the time” or “never”). These questions, which were adapted from the 5-item Mental Health Inventory (MHI-5) screening test, were used to calculate a mental health composite (MHC) score [18]. We asked 13 questions about availability of personal social support with each response rated on a 5-point Likert scale (ranging from 1 being “none of the time” to 5 being “all of the time”). These questions incorporate four social support scales (emotional/informational, tangible, positive interaction, affectionate), and were used to calculate the Social Support Index (SSI), from the Medical Outcomes Study [19]. Both the MHC and SSI scores were independently averaged and then transformed to scores ranging from 1 to 100, with higher numbers signifying better emotional health and more social support. We asked participants if they had gone without food, clothing, or housing (“basic necessities”) during the prior 6 months because they needed money for healthcare, or if they had foregone healthcare during the prior 6 months because they needed money for food, clothing, or housing [20].

After completing the survey, participants were offered free HIV counseling and testing, and HIV-infected participants were offered free CD4 count testing. Those who tested positive for HIV were referred for appropriate care and treatment. All HIV testing, care, and treatment was provided in accordance with current South African Department of Health HIV testing and treatment guidelines [3].

### Statistical analyses

Late-stage HIV disease presentation was defined as a CD4 count <100 cells/mm^3^ at the time of initial HIV diagnosis. To allow incorporation of the perceived barriers into multivariate models, while also minimizing possible collinearity, we categorized the 12 barriers into 5 groups (service delivery, financial, personal health perception, logistical, and structural). Service delivery barriers included “have to wait too long to see the nurse/doctor,” “the nurse/doctor does not speak my language,” and “not treated with respect by the nurse/doctor.” Financial barriers included “could not afford medications” and “could not afford the cost of transportation.” Personal health perception barriers included “didn't think it was necessary, because didn't feel sick” and “felt too sick.” Logistical barriers included “could not get time off work” and “had to take care of someone else.” Structural barriers included “could not get to the clinic during the hours it was open,” “could not arrange transport to the clinic,” and “did not know where to find care.” Poor emotional health and poor social support were defined as an MHC and SSI score below the median value, respectively.

We used Chi-squared and Fisher's Exact tests to compare potential risk factors between those presenting with and without late-stage HIV disease. We used an iterative model building approach to construct a series of logistic regression models to identify factors associated with late-stage disease presentation. First, we used bivariate logistic regression models to determine odds ratios (OR) of presenting with late-stage HIV disease. To build a multivariate logistic regression model and generate adjusted odds ratios (AOR), we included age, sex, and any variable with a p-value <0.15 in bivariate analyses into a single model. We then removed one variable at a time for those variables with a p-value >0.15, and after each variable was removed, the model was refit to evaluate the remaining variables. Finally, variables not selected based on the initial unadjusted analyses were included in the multivariate model to assess their significance in the presence of other variables, and all variables were retained if they had p-values <0.15. To minimize the potential for collinearity, we assessed the correlation between all pairs of independent variables and verified that no pair of variables included in the same regression model was highly correlated with a Spearman r >0.60. All reported p-values were two-tailed, and a p-value <0.05 was considered statistically significant. We conducted analyses using SAS software (version 9.2; SAS Institute, Cary, NC).

## Results

### Cohort characteristics

Among 3,669 people screened for the study, 2,694 met eligibility criteria and enrolled in the study. Among those enrolled, 1,026 (38.1%) tested positive for HIV, of which 830 adults (80.9% of HIV-infected participants) completed both CD4 testing and the study survey. Among those, the median CD4 count was 186 cells/mm^3^ (interquartile range 70–345 cells/mm^3^), and 279 (33.6%) participants had late-stage HIV disease at the time of their initial HIV diagnosis.

Within the cohort, 249 (30.0%) were >40 years of age, 415 (50.0%) were male, and 449 (54.1%) had not completed high school (Table 1). Over half (56.5%) worked outside the home, and 688 (82.9%) participants reported living ≥5 kilometers from the clinic. Most participants (79.7%) had never previously been tested for HIV, including 54 of the 60 participants (90.0%) who had spent an overnight in a hospital during the prior year. Reported HIV testing of participants hospitalized during the prior year (6/60 or 10.0%) was significantly lower than the reported HIV testing among participants who had not been hospitalized during the prior year (162/769 or 21.1%) (p = 0.04).

**Table 1:** Characteristics of HIV-infected adults with and without late-stage disease (CD4<100 cells/mm^3^) at the time of initial HIV diagnosis in a study of HIV testing in South Africa (N = 830).

Variable	Total Cohort N (%)	Presented with late-stage HIV disease N (%)	Presented without late-stage HIV disease N (%)	p-value
**Demographics**				
Age ≥40 years	249 (30.0)	95 (34.1)	154 (27.9)	0.070
Age <40 years	581 (70.0)	184 (65.9)	397 (72.1)	
Male	415 (50.0)	159 (57.0)	256 (46.5)	0.004
Female	415 (50.0)	120 (43.0)	295 (53.5)	
**Education**				
Did not complete high school	449 (54.1)	162 (58.1)	287 (52.1)	0.103
Did complete high school	381 (45.9)	117 (41.9)	264 (47.9)	
**Marital Status**				0.137
Never married	666 (80.2)	220 (78.9)	446 (80.9)	
Currently married	123 (14.8)	41 (14.7)	82 (14.9)	
Divorced/separated	13 (1.6)	3 (1.1)	10 (1.8)	
Widowed	28 (3.4)	15 (5.4)	13 (2.4)	
**If not married, in current relationship?**				0.643
No	226 (32.0)	80 (33.6)	146 (31.1)	
Yes, <6 months	84 (11.9)	25 (10.5)	59 (12.6)	
Yes, ≥6 months	397 (56.2)	133 (55.9)	264 (56.3)	
**Employment**				
Currently working outside home	469 (56.5)	168 (60.2)	301 (54.6)	0.125
Not currently working outside home	361 (43.5)	111 (39.8)	250 (45.4)	
Working <20 hours outside home	32 (6.8)	18 (10.7)	14 (4.7)	0.013
Working ≥20 hours outside home	437 (93.2)	150 (89.3)	287 (95.3)	
**Proximity to the HIV Clinic**				
Distance to clinic ≥5 kilometers	688 (82.9)	257 (92.1)	431 (78.2)	<0.0001
Distance to clinic <5 kilometers	142 (17.1)	22 (7.9)	120 (21.8)	
Travel time to clinic ≥30 minutes	303 (36.5)	107 (38.4)	196 (35.6)	0.432
Travel time to clinic <30 minutes	527 (63.5)	172 (61.6)	355 (64.4)	
**Health Care Usage**				
No prior HIV testing	661 (79.7)	237 (85.0)	424 (77.1)	0.008
Any prior HIV testing	168 (20.3)	42 (15.0)	126 (22.9)	
Any overnight hospital stay in last year	60 (7.2)	27 (9.7)	33 (6.0)	0.053
No overnight hospital stay in last year	770 (92.8)	252 (90.3)	518 (94.0)	
**Emotional Health and Social Support**				
Poor Emotional Health (< median value)	399 (48.1)	157 (56.3)	242 (43.9)	<0.001
Good Emotional Health (≥ median value)	431 (51.9)	122 (43.7)	309 (56.1)	
Poor Social Support (< median value)	416 (50.1)	163 (58.4)	253 (45.9)	<0.001
Good Social Support (≥ median value)	414 (49.9)	116 (41.6)	298 (54.1)	
**Competing Needs to Healthcare**				
Gone without healthcare because needed money for food, clothing, or housing	227 (27.3)	90 (32.3)	137 (24.9)	0.024
Never gone without healthcare because needed money for food, clothing, or housing	603 (72.6)	189 (67.7)	414 (75.1)	
Foregone food, clothing, or housing because needed money for healthcare	196 (23.6)	75 (26.9)	121 (22.0)	0.115
Never foregone food, clothing, or housing because needed money for healthcare	634 (76.4)	204 (73.1)	430 (78.0)	
**Study Site**				0.001
McCord Hospital	437 (52.7)	168 (60.2)	269 (48.8)	
St. Mary's Hospital	281 (33.9)	88 (31.5)	193 (35.0)	
Municipal Clinics	112 (13.5)	23 (8.2)	89 (16.2)	

### Competing needs to healthcare

**Figure 1:**
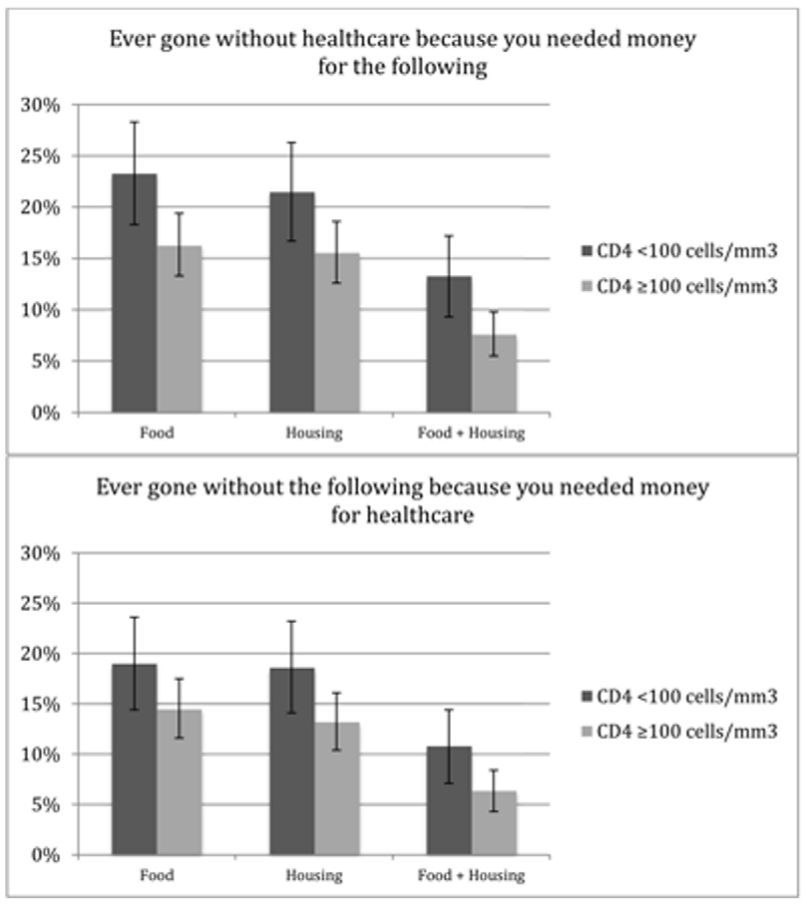
Competing needs to receiving healthcare among those with and without late-stage disease (CD4<100 cells/mm^3^) at the time of initial HIV diagnosis (N = 830). Error bars represent 95% confidence intervals.

Overall, 227 (27.3%) participants had ever gone without healthcare because they needed money for basic necessities (food, clothing, or housing). In bivariate analysis, this was more common among those presenting with late-stage HIV disease (p = 0.02). Participants who presented with late-stage disease were also more likely to have gone without healthcare to pay for food (23.3% vs. 16.3%, p = 0.02), housing (22.5% vs. 15.6%, p = 0.04), and food and housing (13.3% vs. 7.6%, p = 0.009) (Figure 1, top). Similarly, 196 participants (23.6%) had ever foregone basic necessities because they needed money for healthcare. Participants who presented with late-stage disease were more likely to have foregone housing (18.6% vs. 13.2%, p = 0.04), and food and housing (10.8% vs. 6.4%, p = 0.03) to pay for healthcare (Figure 1, bottom).

### Perceived barriers to medical care

The most commonly reported perceived barriers to medical care were “have to wait too long to see the nurse or doctor” (31.4%), “could not afford medications” (26.0%), and “didn't think it was necessary, because didn't feel sick” (24.7%) (Table 2). Participants who reported a perceived barrier of “felt too sick” had a 2.97-fold higher odds (95% CI 2.00–4.41) of late-stage HIV disease presentation. Perceived barriers of “could not afford the cost of transportation” (OR 1.80, 95% CI 1.29–2.29), “could not afford medications” (OR 1.79, 95% CI 1.30–2.46), “could not arrange transport to the clinic” (OR 1.71, 95% CI 1.20–2.43), “have to wait too long to see the nurse/doctor” (OR 1.49, 95% CI 1.10–2.02), and “could not get to the clinic during the hours it was open” (OR 1.46, 95% CI 1.04–2.05) were also more commonly reported among those presenting with late-stage HIV disease.

**Table 2:** Perceived barriers to medical care among those with and without late-stage disease (CD4<100 cells/mm^3^) at the time of initial HIV diagnosis (N = 830).

Perceived Barrier	Total Cohort N (%)	Presented with late-stage HIV disease N (%)	Presented without late-stage HIV disease N (%)	p-value	Odds Ratio (95% CI)
Have to wait too long to see the nurse/doctor	261 (31.4)	104 (37.3)	157 (28.5)	0.010	1.49 (1.10–2.02)
Could not afford medications	216 (26.0)	94 (33.7)	122 (22.1)	<0.001	1.79 (1.30–2.46)
Didn't think it was necessary, because didn't feel sick	205 (24.7)	68 (24.4)	137 (24.9)	0.877	0.97 (0.70–1.36)
Could not afford the cost of transportation	198 (23.9)	87 (31.2)	111 (20.1)	<0.001	1.80 (1.29–2.49)
Could not get to the clinic during the hours it was open	183 (22.1)	74 (26.5)	109 (19.8)	0.027	1.46 (1.04–2.05)
Could not arrange transport to the clinic	158 (19.0)	69 (24.7)	89 (16.2)	0.003	1.71 (1.20–2.43)
Could not get time off work	135 (16.3)	49 (17.6)	86 (15.6)	0.471	1.15 (0.78–1.69)
Felt too sick	120 (14.5)	67 (24.0)	53 (9.6)	<0.001	2.97 (2.00–4.41)
Did not know where to find care	120 (14.5)	37 (13.3)	83 (15.1)	0.486	0.86 (0.57–1.31)
Had to take care of someone else	104 (12.5)	43 (15.4)	61 (11.1)	0.074	1.46 (0.96–2.23)
The nurse/doctor does not speak my language	71 (8.6)	26 (9.3)	45 (8.2)	0.575	1.16 (0.70–1.92)
Were not treated with respect by the nurse/doctor	67 (8.1)	27 (9.7)	40 (7.3)	0.227	1.37 (0.82–2.28)

### Factors associated with late-stage HIV disease presentation

In the multivariate logistic regression model (Table 3), factors associated with presentation to care with late-stage HIV disease were living ≥5 kilometers from the clinic, having gone without healthcare because money was needed for basic necessities, being male, working outside the home, having a perception of health service delivery barriers, and poor emotional health. Living ≥5 kilometers from the clinic conferred a 2.80-fold (95% CI 1.68–4.67) higher odds of presenting with late-stage HIV disease. Having gone without healthcare because money was needed for basic necessities (AOR 1.67, 95% CI 1.17–2.37), being male (AOR 1.66, 95% CI 1.22–2.26), and working outside the home (AOR 1.48, 95% CI 1.07–2.05) had higher odds of late-stage HIV disease presentation. Among the categories of perceived barriers to medical care, only a perception of barriers related to health service delivery (AOR 1.48, 95% CI 1.07–2.05), which included “have to wait too long to see the nurse/doctor”, was significantly associated with late-stage HIV disease presentation in multivariate analyses. Finally, while both poor emotional health and poor social support were significant in bivariate analyses, only poor emotional health (AOR 1.41, 95% CI 1.03–1.94) was significant in the multivariate model.

**Table 3:** Bivariate and multivariate logistic regression models for risk of late-stage HIV disease (CD4<100 cells/mm^3^) at the time of initial HIV diagnosis (N = 830).

	Odds Ratio (95% CI)	p-value	Adjusted Odds Ratio (95% CI)	p-value
**Demographic**				
Age <40 years	Reference	–	Reference	–
Age ≥40 years	1.33 (0.98–1.81)	0.070	1.07 (0.77–1.48)	0.701
Female	Reference	–	Reference	–
Male	1.53 (1.14–2.04)	0.004	1.66 (1.22–2.26)	0.001
**Education**				
Completed high school	Reference	–	–	–
Did not complete high school	1.27 (0.95–1.70)	0.103	–	–
**Employment**				
Not currently working outside home	Reference	–	Reference	–
Currently working outside home	1.26 (0.94–1.68)	0.125	1.48 (1.07–2.05)	0.019
Working ≥20 hours outside home	Reference	–	–	–
Working <20 hours outside home	2.46 (1.19–5.08)	0.015	–	–
**Proximity to the HIV Clinic**				
Distance to clinic <5 kilometers	Reference	–	Reference	–
Distance to clinic ≥5 kilometers	3.25 (2.01–5.26)	<0.001	2.80 (1.68–4.67)	<0.001
Travel time to clinic <30 minutes	Reference	–	–	–
Travel time to clinic ≥30 minutes	1.13 (0.84–1.52)	0.432	–	–
**Health Care Usage**				
Prior HIV testing	Reference	–	–	–
No prior HIV testing	1.68 (1.14–2.46)	0.008	–	–
No overnight hospital stay in last year	Reference	–	Reference	–
Any overnight hospital stay in last year	1.68 (0.99–2.86)	0.055	1.64 (0.94–2.88)	0.084
**Emotional Health and Social Support**				
Good Emotional Health (≥ median value)	Reference	–	Reference	–
Poor Emotional Health (< median value)	1.64 (1.23–2.20)	0.001	1.41 (1.03–1.94)	0.034
Good Social Support (≥ median value)	Reference	–	Reference	–
Poor Social Support (< median value)	1.66 (1.24–2.21)	0.001	1.35 (0.97–1.88)	0.072
**Perceived Barriers to Medical Care**				
No service delivery barriers	Reference	–	Reference	–
Service delivery barriers	1.57 (1.16–2.11)	0.003	1.48 (1.07–2.05)	0.018
No financial barriers	Reference	–	–	–
Financial barriers	1.89 (1.38–2.58)	<0.001	–	–
No personal health barriers	Reference	–	–	–
Personal health barriers	1.36 (1.00–1.84)	0.049	–	–
No logistical barriers	Reference	–	–	–
Logistical barriers	1.39 (1.00–1.94)	0.049	–	–
No structural barriers	Reference	–	–	–
Structural barriers	1.34 (0.99–1.82)	0.060	–	–
**Competing Needs to Health Care**				
Never gone without healthcare because needed money for food, clothing, or housing	Reference	–	Reference	–
Gone without healthcare because needed money for food, clothing, or housing	1.44 (1.05–1.98)	0.024	1.67 (1.17–2.37)	0.004
Never foregone food, clothing, or housing because needed money for healthcare	Reference	–	–	–
Foregone food, clothing, or housing because needed money for healthcare	1.31 (0.94–1.82)	0.115	–	–

## Discussion

As both guidelines and data increasingly support earlier HIV treatment, it is imperative to understand why patients continue to present with advanced HIV. In a large cohort of outpatient clinic attendees newly diagnosed with HIV in South Africa, the main risk factors for presenting with late-stage HIV disease were living further from the clinic, being male, and having gone without healthcare to pay for basic living necessities. Other variables associated with late-stage disease presentation were working outside the home, having a perception of barriers to health service delivery, such as long wait times, and having poor emotional health. These findings provide focused targets for improving HIV testing programs in order to diagnose people earlier and reduce the number of adults presenting to care with late-stage HIV disease.

Several studies have examined risk factors for late-stage disease presentation in sub-Saharan Africa. In Uganda, studies by Kigozi et al. and Wanyenze et al. found significant risk factors were being male, older, and having no secondary education, similar to our findings [21], [22]. They additionally found that people receiving healthcare in a non-medical setting (pharmacy, home, or by traditional healer) or having many sexual partners, both of which were variables we did not obtain, were more likely to present with late-stage disease. In Ethiopia, which has a very different cultural population than South Africa, non-pregnant women, frequent alcohol users, those in a long-term relationship, and people who perceived ART to have many side effects or HIV as a stigmatizing disease were more likely to present with late-stage disease [23]. In our cohort, relationships longer than 6 months had no impact on late-stage presentation, and we did not assess alcohol use or perception of HIV as a stigmatizing disease. The current study is unique from these previous studies by assessing distance to the clinic, perceived barriers to healthcare, competing needs to healthcare, and emotional health and social support structures, before participants were aware of their HIV-infected status.

In our cohort, several structural barriers, such as longer distance to the clinic, a perception of poor service delivery, and working outside the home, were among the strongest risk factors for late-stage HIV disease. Other studies in Africa have shown similar structural barriers, such as longer distances and higher transportation costs, to be related to loss-to-care before ART initiation [24]–[28]. One potential approach to ensuring better service delivery and earlier HIV diagnosis could be the use of active, mobile HIV testing strategies [29]–[31].

Despite the frequent occurrence of late-stage HIV disease presentation in sub-Saharan Africa, there has been an incomplete and inconsistent understanding of the perceived personal barriers to HIV testing [32]. In Botswana, perceived barriers to HIV testing included fear of learning one's status, lack of perceived HIV risk, and fear of having to change sexual practices if positive [33]. In Ethiopia, a perception of HIV as a highly stigmatizing disease was common among people who presented with late-stage disease [23]. Although we did not assess HIV-related stigma, our findings indicate that a perception of service delivery, structural, or financial barriers are obstacles that prevented people from learning their HIV-infected status.

Over one-quarter of those in our cohort reported having gone without healthcare because they needed money for basic necessities, and they were more likely to present with late-stage disease. In several sub-Saharan African studies, food insecurity has been associated with poor ART adherence, more opportunistic infections, missed clinic visits, and increased hospitalizations [34]–[36]. Food insecurity is more common among older, unmarried, HIV-infected adults [34], and thus food insecurity should be addressed as part of comprehensive HIV treatment programs in resource-limited settings [37]. While our findings support the observed negative effects of food insecurity, our results suggest that housing insecurity is also a common problem and associated with late-stage HIV disease presentation.

We found that poor emotional health and social support, both of which were assessed before HIV testing, were associated with a higher likelihood of presentation with late-stage HIV. Depression is common among HIV-infected adults [38], [39], and mental health problems are vastly undertreated in resource-poor settings [40], [41]. Rates of depression are also higher in symptomatic HIV-infected people [42]. While little data exists on outcomes among HIV-infected adults with mental health issues in developing countries, one U.S.-based study found higher rates of mortality among HIV-infected adults with depression [43]. Studies from Uganda and Ethiopia have reported conflicting results about whether poor mental health influences ART adherence [44], [45]. While evidence has been lacking on the association between mental health disorders and uptake of diagnostic and treatment services for HIV [40], our findings suggest that mental health issues and poor social support structures may be important risk factors for delayed HIV testing.

In our study, which excluded known HIV-infected adults, the 60 participants who had been hospitalized during the prior year represented important missed opportunities for HIV testing. Surprisingly, self-reported prior HIV testing was less common among those who had been hospitalized, since South African guidelines recommend “offering HIV testing to all clients attending health-care facilities as a standard component of medical care, unless the client actively refuses” [46]. Our finding suggests that either providers were not adherent to national guidelines or hospitalized patients refused HIV testing at very high rates (90%). Regardless, those missed opportunities represented 7.2% of our cohort, suggesting that HIV testing of hospitalized patients remains suboptimal.

Our findings were primarily limited by the accuracy of self-reported survey data. In this cross-sectional survey, we were not able to determine causality with associations and the analysis was not designed to fully examine the social stigma of HIV as a barrier to testing. We used a definition of late-stage HIV disease of CD4<100 cells/mm^3^, which portends a major risk for cryptococcal meningitis, a leading cause of AIDS-related deaths in sub-Saharan Africa [47]. This definition allowed us to identify risk factors and perceived barriers among the people with the greatest risk for severe opportunistic infections and mortality. While we did not assess some variables shown to be associated with late-stage presentation in other African studies (alcohol consumption, number of sexual partners, or ownership of material goods), we examined many important measures not studied in other studies of late-stage HIV disease presentation in sub-Saharan Africa. Finally, a small number of HIV-infected participants either did not fully complete our survey or agree to CD4 count testing, so we cannot assess their influence on our findings.

In conclusion, we found independent risk factors for presentation with late-stage HIV disease from diverse domains, including geographic, economic, demographic, social, and psychosocial. These can directly inform efforts to improve HIV testing. Such efforts should focus on various interventions, such as the use of active mobile testing strategies, financial assistance, or providing food supplementation, to reduce late-stage disease presentation in resource-poor settings (Table 4). Simply expanding HIV testing will not ensure ART-eligible people are enrolled in care or initiated on ART; innovative approaches are also needed to improve subsequent linkage to treatment [48], [49]. Providing increased and targeted HIV testing to those at greatest risk for late-stage HIV disease, and subsequently linking them to HIV care should reduce AIDS-related morbidity and mortality.

**Table 4:** Identified barriers and possible solutions to reduce the risk of late-stage HIV disease presentation.

Identified Barriers	Possible Solutions
• Living >5 kilometers from the test site	• Provide mobile HIV testing services
• Reported competing needs to healthcare	• Provide financial assistance to those at risk
• Worked outside the home	• Offer periodic HIV screening at employment and business locations
• Perceived health service delivery barrier	• Expand clinic HIV testing hours to include nights and weekends• Provide travel vouchers to those presenting for HIV testing
• Poor emotional health	• Increase depression screening in the community• Strengthen integration of mental health services into primary health care
